# Strain Structure and Dynamics Revealed by Targeted Deep Sequencing of the Honey Bee Gut Microbiome

**DOI:** 10.1128/mSphere.00694-20

**Published:** 2020-08-26

**Authors:** Louis-Marie Bobay, Emily F. Wissel, Kasie Raymann

**Affiliations:** a Department of Biology, University of North Carolina at Greensboro, Greensboro, North Carolina, USA; Clemson University

**Keywords:** community dynamics, honey bee, microbiome, strain diversity

## Abstract

Host-associated microbiomes can be critical for the health and proper development of animals and plants. The answers to many fundamental questions regarding the modes of acquisition and microevolution of microbiome communities remain to be established. Deciphering strain-level dynamics is essential to fully understand how microbial communities evolve, but the forces shaping the strain-level dynamics of microbial communities remain largely unexplored, mostly because of methodological issues and cost. Here, we used targeted strain-level deep sequencing to uncover the strain dynamics within a host-associated microbial community using the honey bee gut microbiome as a model system. Our results revealed that amplicon sequencing of conserved protein-coding gene regions using species-specific primers is a cost-effective and accurate method for exploring strain-level diversity. In fact, using this method we were able to confirm strain-level results that have been obtained from whole-genome shotgun sequencing of the honey bee gut microbiome but with a much higher resolution. Importantly, our deep sequencing approach allowed us to explore the impact of low-frequency strains (i.e., cryptic strains) on microbiome dynamics. Results show that cryptic strain diversity is not responsible for the observed variations in microbiome composition across bees. Altogether, the findings revealed new fundamental insights regarding strain dynamics of host-associated microbiomes.

**IMPORTANCE** The factors driving fine-scale composition and dynamics of gut microbial communities are poorly understood. In this study, we used metagenomic amplicon deep sequencing to decipher the strain dynamics of two key members of the honey bee gut microbiome. Using this high-throughput and cost-effective approach, we were able to confirm results from previous large-scale whole-genome shotgun (WGS) metagenomic sequencing studies while also gaining additional insights into the community dynamics of two core members of the honey bee gut microbiome. Moreover, we were able to show that cryptic strains are not responsible for the observed variations in microbiome composition across bees.

## Abstract (precis)

The factors driving fine-scale composition and dynamics of gut microbial communities are poorly understood. In this study, we used metagenomic amplicon deep sequencing to decipher the strain dynamics of two key members of the honey bee gut microbiome. Using this high-throughput and cost-effective approach, we were able to confirm results from previous large-scale whole-genome shotgun (WGS) metagenomic sequencing studies while also gaining additional insights into the community dynamics of two core members of the honey bee gut microbiome. Moreover, we were able to show that cryptic strains are not responsible for the observed variations in microbiome composition across bees.

## INTRODUCTION

There is increasing evidence that deciphering strain-level diversity is crucial for understanding the impact of microbiomes on host health. However, the fine-scale community dynamics within host microbiomes is still poorly characterized and understood. Our lack of understanding of host-associated microbial community dynamics can be attributed to two main factors. (i) Most methodologies lack the accuracy to fully evaluate strain-level diversity. (ii) Host-associated microbial communities are generally very complex, making it challenging to disentangle strain-level dynamics (1, 2). A common method used in microbiome studies is amplicon sequencing of the 16S rRNA gene, which fails to provide an accurate picture of species, and especially strain-level, diversity within a community. Whole-genome shotgun (WGS) metagenomic sequencing can provide insights about strain diversity. However, WGS metagenomics requires a high sequencing depth to recover strain diversity, making it expensive to apply to large numbers of samples and virtually incapable of capturing low-frequency species and strains, especially in complex communities. An alternative but rarely used method for assessing strain-level diversity employs amplicon sequencing of species-specific protein-coding gene markers (3–6), similar to multilocus sequence typing (MLST) but applied to natural communities. We will refer to this method as metagenomic amplicon strain typing (MAST). By assessing nucleotide variation in core protein-coding genes, MAST can identify strain diversity for each individual species in a community. Therefore, this approach can be used to determine the population structure and dynamics of different microbial strains across samples, locations, times, and conditions. However, one limitation of this approach is that it requires specific primers for each species of the community. Therefore, it cannot easily be applied to entire complex communities or to species for which reference genomes are not available.

We used the honey bee (Apis mellifera) as a model system to study the population dynamics of host-associated microbiomes using the MAST method. The honey bee is an ideal model system for tackling fundamental questions about microbial community dynamics because its microbiome is simple (eight species make up ∼95% of the community), conserved (five core species are found in all honey bees globally), and vertically transmitted (from bee to bee) (7–10). Despite the simplicity and conservation of the honey bee gut microbial community at the species level, a large amount of strain-level variation has been shown to exist within each of the core species (4, 11–13). The strain-level diversity within honey bees and bumble bees has been investigated mostly through WGS metagenomics and isolate or single-cell sequencing (11, 12, 14–20), though one study used the MAST method to investigate strain-level diversity of Snodgrassella alvi in honey bees and bumble bees (4). From these studies, it has been shown that individual bees from the same colony harbor different strains, resulting in very high strain-level diversity within a population, i.e., colony (4, 11–13). There is evidence that strains can be dominant in an individual bee and absent from another within the same colony (4, 12) and that within an individual, closely related strains generally coexist together (12). The high strain-level diversity does not seem to correspond to variation in age or sampling time, and strains are not unique to geographic location, e.g., the same strains have been found in bees from different countries (4, 12). Moreover, strains within the same species often possess different metabolic capabilities, making individual communities functionally distinct (14–16, 20–22). Although WGS metagenomic studies have revealed a lot about strain-level dynamics in the honey bee gut, they were unable to rule out the possibility that low-frequency variants within the population could explain the strain-level population dynamics. Likewise, the previous study which used the MAST method to investigate strain diversity designed their marker (*MinD*) to capture *Snodgrassella* diversity across both bumble bees and honey bees (4), limiting their ability to capture fine-scale strain variation because of the high level of divergence of *Snodgrassella*, which is likely composed of multiple species across these different bee hosts.

Here, we used MAST on protein-coding gene markers for two of the most dominant core members of the honey bee gut microbiome, Snodgrassella alvi and *Gilliamella* spp., to evaluate strain composition in four honey bee colonies from four different locations in the United States. We were able to confirm the results of previous strain-level studies while also gaining additional insights. Importantly, we were able to explore the impact of low-frequency variants on microbiome dynamics and show that cryptic strain diversity is not responsible for the observed variations in microbiome composition across bees. We identified that strain composition is far from random in honey bees, where several strains are frequently associated together, while others almost never co-occur in the same host. We found that many bees from the same hive harbor highly divergent strain compositions, while bees sampled across different geographic locations (different states in the United States) frequently harbor similar strain communities. Strains present within an individual honey bee typically possess very high sequence similarity, and our results indicate that they did not originate from within-host diversification.

## RESULTS

### Analysis of the MAST markers.

We analyzed the strain composition of two core members of the honey bee gut microbiome: S. alvi and *Gilliamella* spp. Honey bees were sampled from a single colony from four different locations in the United States (numbers of sampled bees per location, 103 for Texas, 21 for Tennessee, 9 for Utah, and 11 for Florida). DNA was extracted from the gut, and amplicon sequencing was performed for four markers (*guaA* and g*luS* for *S. alvi* and *pflA* and *rimM* for *Gilliamella* spp.) on each individual bee (6). These markers were designed to target only *Snodgrassella* and *Gilliamella* in honey bees (*A. mellifera*), since creating markers that also capture bumble bee (*Bombus* spp.) strains resulted in a lack of resolution (6).

Sequenced markers were quality filtered to avoid overprediction due to sequencing errors (see Materials and Methods). Sequences were aligned, and strains were considered different if they possessed ≥1 nucleotide difference along the marker. The coverage depth of each marker (average of 46,000 reads per marker and per sample) allowed us to identify strains with high confidence (see Materials and Methods and Table S1 and Fig. S1 in the supplemental material). Using this approach, we identified more than 200 different strains for each marker across all bees analyzed.

10.1128/mSphere.00694-20.1 FIG S1 Average read coverage for each marker by sampling location. Download FIG S1, PDF file, 0.2 MB. Copyright © 2020 Bobay et al. 2020 Bobay et al. This content is distributed under the terms of the Creative Commons Attribution 4.0 International license.

10.1128/mSphere.00694-20.8 TABLE S1 Total number of reads obtained for each sample for each marker. NA represents samples that reads were not obtained due to issues with DNA extraction or sequencing. Download Table S1, XLSX file, 0.01 MB. Copyright © 2020 Bobay et al. 2020 Bobay et al. This content is distributed under the terms of the Creative Commons Attribution 4.0 International license.

We also built the core genome phylogenies and marker sequence phylogenies (amplicon region of each gene used for MAST) using all publicly available genomes of *S. alvi* and *Gilliamella* spp. This allowed us to determine whether the marker sequence phylogenies were congruent with the species phylogenies. The resolution of the marker sequence phylogenies is low due to the limited number of base pairs (less than 500 bp for each marker), but overall, all four marker phylogenies were consistent with the core genome phylogenies (Fig. S2).

10.1128/mSphere.00694-20.2 FIG S2 Phylogenetic trees of *A. mellfiera*-associated *S. alvi* and *Gilliamella* strains available on NCBI. (A) Core genome reference phylogeny of *S. alvi*, (B) MAST *gluS* marker phylogeny, and (C) MAST *guaA* marker phylogeny. (D) Core genome reference phylogeny, (E) MAST *rimM* marker phylogeny, and (F) MAST *pflA* marker phylogeny. Blue shading is used to delineate the different *Gilliamella* species or phylotypes. Phylogenetic trees were constructed with RAxML v8 with a GAMMA + GTR model. Download FIG S2, PDF file, 0.3 MB. Copyright © 2020 Bobay et al. 2020 Bobay et al. This content is distributed under the terms of the Creative Commons Attribution 4.0 International license.

### Strain diversity of *S. alvi* and *Gilliamella* spp.

Since designing our MAST markers (6), the number of sequenced genomes for *Gilliamella* has more than doubled and *Gilliamella* has been split into at least two, possibly three, distinct species (12, 23). Thus, we first checked to see whether our four markers captured the entire strain diversity of *S. alvi* and *Gilliamella* spp. To this aim, we built the phylogeny for each marker using our MAST-derived sequences and the corresponding sequences of the publicly available genomes. Our results indicate that both markers *gluS* and *guaA* captured all the diversity of sequenced *S. alvi* from A. mellifera (Fig. S3), which is thought to be the sole *Snodgrassella* species present in *A. mellifera* (12). In contrast, our phylogenetic analysis indicates that the two *Gilliamella* markers offer different resolutions: the *rimM* marker captures strain diversity across all sequenced *Gilliamella* species, whereas *pflA* captures the strain diversity of G. apicola only (Fig. S4). For clarity, the results of the two *Gilliamella* markers will be presented and discussed in parallel, since one marker provides a genus-level resolution of strain diversity and the other depicts the strain diversity within the *G. apicola* species.

10.1128/mSphere.00694-20.3 FIG S3 Phylogenetic tree of *A. mellifera*-associated *S. alvi* strains based on *gluS* and *guaA* MAST markers. The phylogeny was made using the amplified regions of each gene marker from the strains sequenced in this study (green font) as well as the sequenced genomes available on NCBI as of July 2020 (black font and highlighted in green). The trees were constructed with RAxML v8 with a GAMMA + GTR model. Download FIG S3, PDF file, 0.4 MB. Copyright © 2020 Bobay et al. 2020 Bobay et al. This content is distributed under the terms of the Creative Commons Attribution 4.0 International license.

10.1128/mSphere.00694-20.4 FIG S4 Phylogenetic tree of *A. mellifera*-associated *Gilliamella* strains based on *pflA* and *rimM* MAST markers. The phylogeny was made using the amplified regions of each gene marker from the strains sequenced in this study (blue font) as well as the sequenced genomes available on NCBI as of July 2020 (black font and highlighted in blue). The trees were constructed with RAxML v8 with a GAMMA + GTR model. Download FIG S4, PDF file, 0.4 MB. Copyright © 2020 Bobay et al. 2020 Bobay et al. This content is distributed under the terms of the Creative Commons Attribution 4.0 International license.

**FIG 1:**
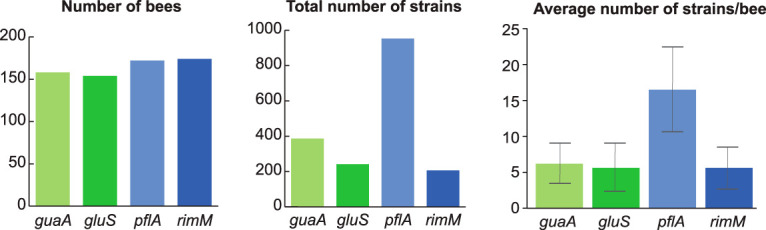
Number of strains identified with the four markers. (Left) Total number of bees analyzed for each marker. (Middle) Total number of strains identified with each marker. (Right) Average number of strains identified per bee. The standard deviations are indicated by error bars. The markers of *S. alvi* and *Gilliamella* spp. are represented in green and blue, respectively.

We identified an average of five to six strains for both *S. alvi* and *Gilliamella* spp. per bee based on three markers (*guaA*, *gluS*, and *rimM*), while the *G. apicola*-specific marker (*pflA*) yielded an average of 16 strains per bee (Fig. 1). For *S. alvi* and *G. apicola*, any two strains were found to differ by seven to nine single nucleotide polymorphisms (SNPs) on average (Fig. S5A). As expected, the *Gilliamella* species marker (*rimM*) displayed higher sequence diversity across strains (∼20 SNPs), due to the fact that it captures multiple species (Fig. S5A).

10.1128/mSphere.00694-20.5 FIG S5 (A) Average number of SNPs across strains estimated with the four gene markers. The markers of *S. alvi* and *G. apicola* are represented in green and blue, respectively. (B) Resampling analysis of strain composition. For each marker, the average number of strains was estimated for different numbers of randomly sampled bees (nonredundant). The *y* axis shows the average number of strains found in 100 combinations of randomly sampled bees. The *x* axis indicates the number of nonredundant resampled bees included in each combination. Download FIG S5, PDF file, 0.2 MB. Copyright © 2020 Bobay et al. 2020 Bobay et al. This content is distributed under the terms of the Creative Commons Attribution 4.0 International license.

We estimated to what extent our procedure was capable of capturing the entire strain diversity of *S. alvi*, *G. apicola*, and *Gilliamella* spp. To do so, we conducted a resampling analysis of the bees used to infer strain diversity and built saturation curves based on each marker (Fig. S5B). Results indicate that a substantial fraction of the strain diversity of these bacteria has been captured by our analysis for the four hives sampled from the four states. However, the saturation curves do not plateau, indicating that we did not capture the entire strain diversity of *Gilliamella* spp. or *S. alvi*. Results likely reflect heterogeneous strain compositions across bees and suggest that a larger strain diversity exists for these two symbionts of the honey bee gut.

### Geography does not drive strain composition of the bee gut microbiome.

**FIG 2:**
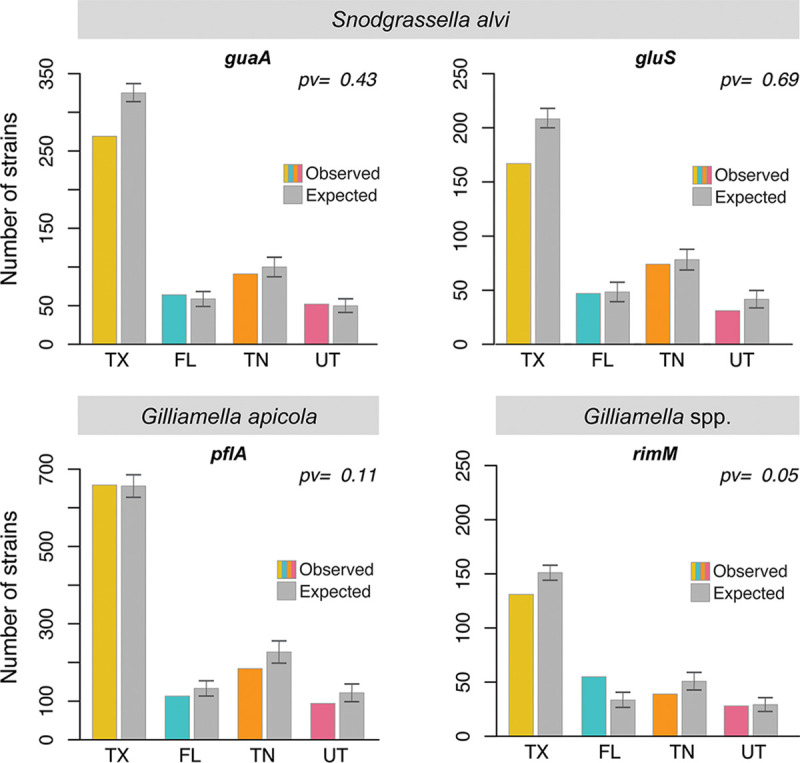
Number of observed strains versus number of randomly expected strains for each location. For each marker, the number of strains is indicated for each location (color bars). The number of strains for each location was compared to the number of strains expected at each location under random expectation (gray bars). The random expectation was obtained by randomly reallocating each bee to a different location. The error bars represent standard deviations. *pv*, *P* value.

We tested whether strain distribution was biased across the hives from the four geographic locations (Texas, Utah, Florida, or Tennessee). We compared the number of distinct strains observed for each location and compared that to the number of expected strains under randomization (i.e., each bee was randomly allocated a state). The number of expected strains under the random distribution was not significantly different from the observed distribution for *S. alvi* and *G. apicola* markers (*P* > 0.1, chi-square test, Fig. 2) but was significant for the *Gilliamella* marker (*P* = 0.005, chi-square test, Bonferroni correction). Overall, these results suggest that strains of *S. alvi* and *G. apicola* are not associated with specific locations and that most strains are likely to be found across the United States.

**FIG 3:**
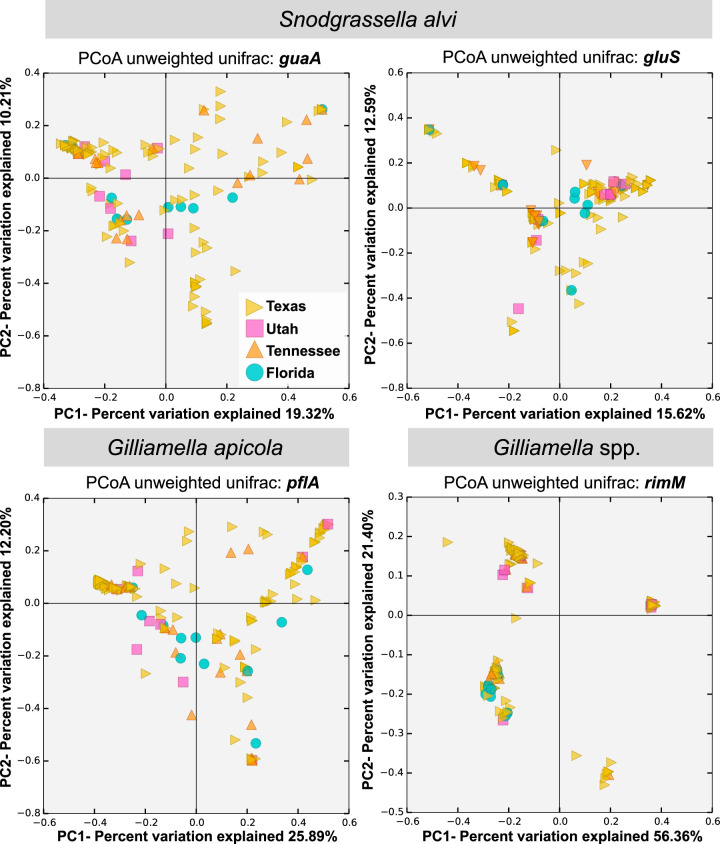
Principal coordinate analyses (PCoA) of strain composition for each bee analyzed based on the four markers and geographic location. Each symbol represents the value for an individual bee. The colors and shapes of the symbols represent the location of each bee (Texas, Utah, Tennessee, or Florida). The analysis was conducted by comparing the composition of strains for all four markers (see Materials and Methods).

In order to further analyze the strain composition (presence/absence) within bees from different hives and locations, we performed principal coordinate analyses using unweighted UniFrac (24) on the four amplicons. The principal coordinate analyses did not show clustering based on hive location for any of the markers or species, indicating that bees from the same hive are not more similar in strain composition than bees sampled from other hives in different states. In contrast, this analysis revealed that some bees sampled from hives at various locations present similar strain compositions (Fig. 3).

### Bacterial strains are not randomly associated within bees.

**FIG 4:**
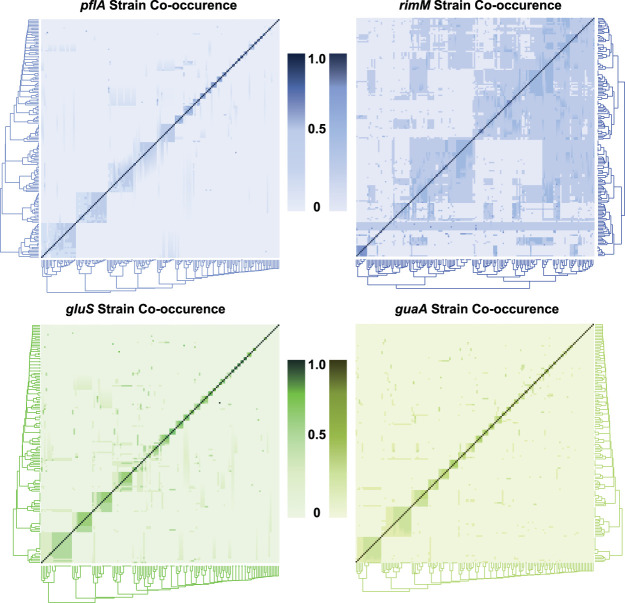
Heatmaps of strain composition across bees for each marker. Each line and column represent a bee. A score of 1.0 represents 100% co-occurrence (the bees are composed of the exact same strains), and 0.0 represents no co-occurrence (the bees do not share any common strains).

To further decipher the distribution of strains across bees and geographic locations, we analyzed the patterns of strain co-occurrence within bees. Consistent with previous findings (12), we observed that several bees tended to present either very similar strain compositions or very different strain compositions (Fig. 4). Similar results were observed when representing the co-occurrence of strains (Fig. S6). For all markers, we found that some strains almost exclusively co-occur together within the same bees while others never or rarely co-occur (Fig. S6). To further test this, we randomly reassorted the strains across bees and counted the number of times the randomly shuffled strains co-occurred. We found that the distribution of strain associations was significantly different from the randomized association for *S. alvi* and *G. apicola* markers (for *guaA*, *gluS*, and *pflA*, *P* < 10^−7^, *P* < 0.003, and *P* < 10^−12^, respectively, chi-square tests with Bonferroni corrections). The test was not found significant for the *Gilliamella rimM* marker (*P* = 0.43, chi-square test).

10.1128/mSphere.00694-20.6 FIG S6 Heatmaps of strain co-occurrence for each marker. Each line and column represent a strain. A score of 1.0 represents 100% co-occurrence (the strains are always found together), and 0.0 represents no co-occurrence (the strains are never found together). Download FIG S6, JPG file, 0.3 MB. Copyright © 2020 Bobay et al. 2020 Bobay et al. This content is distributed under the terms of the Creative Commons Attribution 4.0 International license.

**FIG 5:**
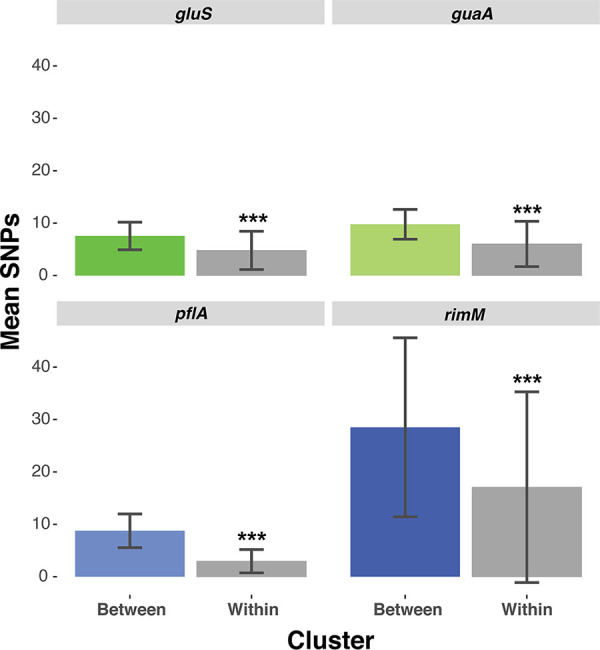
Barplots comparing sequence similarity (mean number of SNPs) between clusters (colored boxes) versus across clusters (gray boxes). All four Wilcoxon tests were significant (***, *P* < 10^−15^).

Overall, we identified around 40 strain clusters (strains that frequently co-occur in the same bees), with each cluster containing approximately three to four strains, with the exception of the *G. apicola* marker *pflA* where a higher strain diversity was captured (Fig. S7A). We found that the vast majority of bees were composed of a single cluster of strains (Fig. S7B). When comparing the strains within each cluster, we found that most strains from the same cluster typically differ by a single SNP or very few SNPs (Fig. S7C). For each of the four markers, strains from the same cluster were more similar to each other than strains from different clusters (*P* < 10^−15^, Wilcoxon test, Fig. 5). Consistent with previous evidence (12), most individual bees were found to possess only a single cluster of closely related strains.

10.1128/mSphere.00694-20.7 FIG S7 (A) Number of strains per cluster. (B) Number of strain clusters per bee. (C) Average number of SNPs within strain clusters. (D) Number of strain clusters obtained for each marker with MCL using different inflation parameters (*I *= 1.2, 2.0, 4.0, and 6.0). The scores used for the clustering measure the co-occurrence of strains across bees (see Materials and Methods). Download FIG S7, PDF file, 0.5 MB. Copyright © 2020 Bobay et al. 2020 Bobay et al. This content is distributed under the terms of the Creative Commons Attribution 4.0 International license.

**FIG 6:**
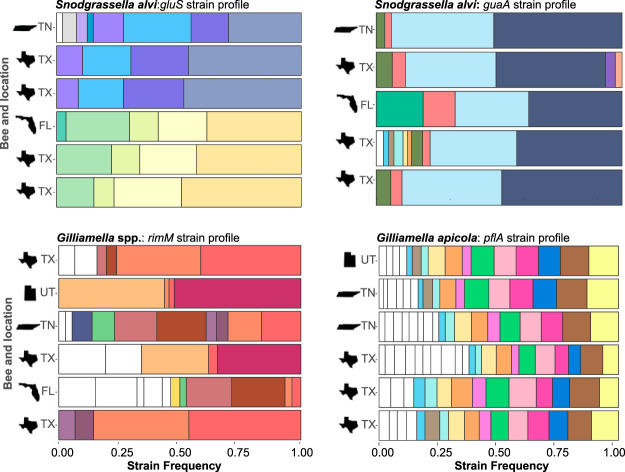
Examples of strain compositions in bees across locations and markers. Each color represents a different strain. Several strains were not shown by colors when too many strains were present in the same graph (*n *> 12).

The fact that bees usually contain a single cluster of strains and that these clusters are composed of closely related strains could indicate that each bee was originally colonized by a single strain which subsequently diversified in its host. However, the distribution of strains across bees rejects this model. Under a model of colonization from a single strain followed by within-host diversification, we would expect that strains evolving within each bee would acquire independent mutations at different sites. Instead, we observed that the exact same strains are often found across hives and locations (Fig. 6), indicating that they did not evolve from a single colonizing strain. This pattern indicates that the multiple closely related strains within a strain cluster are frequently acquired together. Not only do some bees from different locations harbor the same strain clusters, within those clusters, bees from different locations often share the same strains in similar relative abundance (Fig. 6). These results indicate that strain composition and relative abundance are not random because the observed patterns are conserved across bees sampled from remote geographic locations.

We tested whether the strains of *S. alvi* and *Gilliamella* spp. presented specific patterns of interspecies associations. We reasoned that strains from different species living in the same niche might be engaged in specific relationships (e.g., some strains might engage in mutualistic relationships, while others might be antagonists), and this would result in nonrandom associations between the strains of the two species. To test this, we analyzed the patterns of strain co-occurrence based on the four marker pairs: *guaA-pflA*, *guaA-rimM*, *gluS-pflA*, and *gluS-rimM*. As in the previous analysis, we randomly shuffled the strains across bees and estimated whether the association of strains deviated from the random expectation. We did not find any evidence of interspecies association between strains for the four pairs of markers (not significant, chi-square tests), suggesting that interspecies interactions do not result in specific strain associations in our samples.

### Sequencing errors cannot account for the patterns of strain distribution.

We investigated whether PCR amplification artifacts and/or sequencing errors can account for the observed patterns of strain distribution. Indeed, PCR amplification errors can occur during the first amplification cycles and potentially lead to the inference of SNPs at high frequency in the samples (25). Although this process likely leads to an overestimation of inferred strains (when reaching ≥1% frequency), it is unlikely to affect the patterns of strain distribution, i.e., it is unlikely to lead to the exact same sequence variant in different bees. To test this, we simulated the extreme scenario, where the total diversity of strain sequences observed in our data set was introduced exclusively by PCR artifacts. We simulated sequences *in silico* for each of the four markers based on the number of strains detected in each bee. Each strain sequence was generated from the reference sequence by introducing an average number of SNPs based on the average number of SNPs observed between the strains found in one bee following a Poisson process (∼5,000 distinct sequences across markers and samples). Following this procedure, we did not observe a single case of sequence convergence across all samples. These results indicate that, although we might slightly overestimate the number of strains due to PCR errors, this is highly unlikely to account for the patterns of strain distribution across our samples.

### Convergent evolution cannot account for the patterns of strain distribution.

We tested whether selective constraints could account for the patterns of strain distribution observed in this study. Indeed, purifying selection imposes strong constraints on sequence evolution, and one could argue that this could lead to similar sequences by convergent substitutions when sequences are short and many variants are analyzed. We used the relative rates of sequence evolution across codon positions of the four markers to simulate the evolution of sequences, starting from the reference sequence of each marker, while introducing independent mutations until we obtained the total strain diversity captured in our study. The relative rates of codon evolution were determined by the relative allelic diversity across codon positions observed in each of our four markers. As expected for protein-coding genes, we observed that the third codon position was the least constrained by selection; 71 to 82% of the SNPs were observed at this position across markers. The second codon position was the most constrained; 4 to 10% of the SNPs were observed at this position across markers. Finally, the first codon position was found to present an intermediate level of diversity; 12 to 20% of the SNPs were observed at this position across markers. By simulating sequences with these imposed rates of substitution across codon positions, we did not observe a single case of sequence convergence across the simulated sequences, indicating that purifying selection is unlikely to lead to convergent sequence evolution in our markers.

### Cryptic strains do not explain the patterns of strain composition.

**FIG 7:**
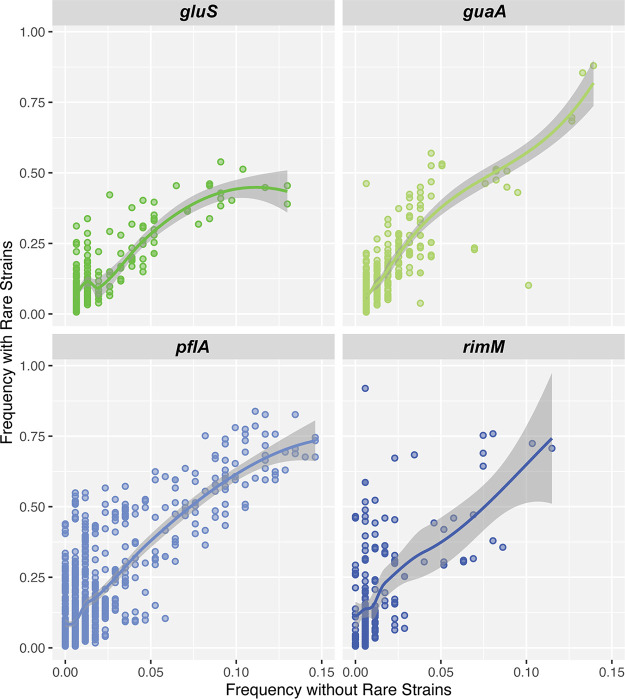
Prevalence of rare strains across bees with relaxed filters. The *x* axis represents the prevalence of each strain in our sample of bees (percentage of bees where the strain has been identified). The *y* axis shows the prevalence of each strain when redefining the strains with relaxed filters: i.e., the strain is considered present even when found <1% frequency in the bee (see Materials and Methods).

The fact that bees from different hives and states present similar strain profiles, whereas many bees from the same hive have completely different strain compositions, suggests that there are complex strain dynamics in the honey bee microbiota. Several hypotheses could explain these patterns (see Discussion). The current data set does not allow us to test all hypotheses; however, we can test the hypothesis that cryptic strain diversity remains present in all bees. One possible explanation of the observed patterns is that each bee could potentially contain a much higher and undetected strain diversity. Thus, our results could be simply due to variations in strain frequencies across bees instead of independent strain acquisitions. All results presented above were generated under stringent filters to avoid overestimation of strain diversity due to sequencing errors, but it is possible that many low-frequency or very low-frequency strains (i.e., cryptic strains) remained unnoticed. Due to the high sequencing depth for each marker, we were able to test this hypothesis. We specifically searched for the presence of our set of identified strains in the set of filtered-out reads in bees where these strains were not detected under our threshold of 1% frequency. For this analysis, a strain was identified even if supported by a single read. We then compared the diversity of strains that would be observed under these loose criteria compared to our original prediction (Fig. 7). As anticipated, we identified the presence of additional strains across a higher proportion of bees. On average, we observed a fivefold increase in the prevalence of each strain across bees, but as mentioned, many likely correspond to sequencing errors. Nevertheless, this procedure did not allow us to recover each strain in every sample (Fig. 7), indicating that most strains are limited to a subset of bee hosts (typically <30%). Of course, we cannot exclude the possibility that additional cryptic strains would be recovered by increasing sequencing depth, but our sequencing depth should allow us, in theory, to detect strains present at 0.00002% frequency on average. Thus, our results support the view that bees present distinct strain compositions and that the distribution of strain clusters is due to mechanisms of transmission and selection, not simply variations in strain frequencies.

## DISCUSSION

We applied a targeted approach to assess the strain-level diversity of the honey bee microbiome. By deep sequencing variable regions of protein-coding genes, we were able to decipher the strain dynamics of two core members of the honey bee microbiome. Although the present study did not attempt to evaluate the diversity of the entire community, we were able to gain additional insights on the strain dynamics of two key members of the honey bee gut microbiome. MAST sequencing allowed us to explore certain hypotheses more thoroughly than would have been possible using shotgun metagenomic sequencing or by sequencing individual isolates. For instance, we were able to search for strains potentially present at frequency as low as 0.00002%. While it is virtually impossible to prove that strains are completely missing from a sample, our results provide higher confidence that cryptic strains are truly absent from our samples and do not account for the observed patterns of strain dynamics.

Overall, our results on strain diversity of *S. alvi* and *Gilliamella* spp. are consistent with previous studies (4, 12). We observed that strain composition within the bee microbiome is not random. Our strain cluster analysis revealed that the same groups of strains frequently co-occur together and that, most of the time, a bee contains only closely related strains. As reported in reference 12, different *Gilliamella* spp. (based on the *rimM* marker) do co-occur in a single bee, but generally only closely related strains within each “species” co-occur. These patterns might be attributed to strain-strain incompatibilities due to resource competition or strain warfare. Alternatively, specific sets of strains could be filtered or selected by the host environment (i.e., the immune system) as previously proposed (26). Interestingly, co-occurring strains typically differed by only a single nucleotide in the marker region. At first, these patterns appeared compatible with a scenario of colonization by a single strain, followed by within-host diversification by mutations. Such a scenario has been observed for some dominant species of the human microbiome (27, 28). However, the consistent presence of the exact same strains across different bees (including bees from different hives and states) indicates that these patterns are not due to within-host diversification. Rather, within-host diversification of a single strain would generate various strains through random mutations affecting different sequence sites (i.e., it is extremely unlikely that random mutations would produce the exact same alleles in multiple bees).

The co-occurrence of strains predominantly composed of closely related strains goes against the intuitive explanation that related strains must compete against one another for the same resources. One possible explanation of these patterns could be due to strain warfare. Bacteria frequently secrete compounds to kill other bacteria but must be immune to their own secreted chemicals such as toxin-antitoxin systems (29, 30), and some strains of *S. alvi* have been shown to encode type VI secretion systems (20). These mechanisms would lead to the elimination of competitors by a single strain; however, recently diverged strains would also encode the same genetic arsenal, and as a result, closely related strains would be immune to the same compounds. As strains diverge from each other over time, their warfare systems would eventually evolve and would not confer cross-immunity. A similar mechanism could be proposed through bacteriophage predation since closely related strains would present similar sensitivities and immunities to bacteriophages (31, 32). Note that in the case of temperate bacteriophages, lysogenic bacteria could encode a prophage themselves, and the occasional reactivation of this prophage could eliminate the nonlysogenic strains, while protecting the prophage-encoding strains (33) (prophages must confer superinfection immunity to be viable [34, 35]). Alternatively, the consistent association of closely related strains could result from the establishment of codependence between strains (i.e., processes similar to the Black Queen hypothesis but limited to closely related strains) (36, 37). For instance, bacteria require the secretion of multiple proteins in the environment to establish biofilms or other structures (38–40). Assuming a simple system with two secreted proteins A and B, the loss of gene A in one clone and the loss of gene B in another clone would establish a situation of codependence between these two newly formed strains. Over time, this situation could potentially lead to the loss by drift of the original strain carrying both gene copies A and B. This scenario of balancing selection could potentially explain why closely related strains are maintained together. This could also explain why the relative frequencies of these strains also appear conserved across sampled bees, since secreted proteins would need to be secreted at similar concentrations to interact with one another efficiently.

Finally, our results showed that bees from the same hive often present dissimilar strain compositions, whereas many bees from different hives and states present similar strain compositions. This observation further suggests that strain composition of the bee gut microbiome is not shaped by neutral processes. We showed that these patterns do not seem compatible with the presence of a higher undetected strain diversity within each bee microbiome (the presence of cryptic strains). However, we cannot completely exclude the possibility that this result could be due to bee migration between hives located in different states or founding queens coming from the same breeders. Commercial hives are often moved across the United States for seasonal field fertilization, and this process could lead to frequent migration of bees across states. Moreover, many bee colonies are founded by sister queens reared by the same operation and shipped with workers. Although we know that the Florida bees were from a commercial colony that was frequently treated with antibiotics and other chemicals, the Tennessee bees were from an organic colony that had not been treated with any chemicals for more than 20 years, the Texas bees were taken from a research colony that had not been treated with any chemicals for more than 2 years, and the Utah bees were sampled from a feral colony that had not been managed for at least 10 years, we lack precise data regarding how these hives were initially founded. Nevertheless, colony migration and the initial founding queens are not likely to account for the observed strain dynamics, since previous studies have demonstrated that the same strains are found in bees from different countries (4, 12). Additionally, age and lifestyle (nurse versus forager) does not seem to be a factor in determining strain composition (12). Thus, previous studies as well as our results support the hypothesis that different strain profiles might be selected by different bee genotypes. A honey bee queen can mate with up to 59 different drones, and on average mates with about 14 drones (41), creating a colony that consists of a wide range of different genotypes. If strain composition is associated with particular genotypes, this could explain the variation within a single hive (26). Finally, these patterns of strain composition could also be driven by colonization order and strain warfare. This scenario implies that the first colonizing strain—which might be random—would prevent the future colonization of certain strains but allow other strains to colonize (42). Because closely related strains are often found associated together, this process would not be driven by niche competition but by direct strain antagonism mediated by warfare systems such as toxin-antitoxin systems, type VI secretion system effectors, and prophages.

One major limitation of the MAST method is the need for sequenced genomes representative of the diversity of each species to develop accurate markers. As previously noted, the markers used in this study were designed using a limited set of genomes (6), which resulted in one of our markers (*rimM*) capturing all honey bee-associated *Gilliamella* species while the other (*pflA*) captured the diversity of only *G. apicola*. Both markers provided useful information, but it is obvious from our results that the marker that captures all *Gilliamella* species comes at the cost of reduced strain resolution, e.g., the *pflA* marker recovered more than 900 strains, while *rimM* recovered only ∼200 strains. Therefore, MAST is a powerful method for analyzing strain diversity, but genome availability and marker design are critical factors in the accuracy of strain detection. Another caveat of using the MAST method is that identical protein-coding amplicon sequences do not necessarily imply that the entire genomes are 100% identical. Therefore, MAST cannot technically determine whether strains are identical across samples. However, the MAST method is a good proxy for strain similarity and composition and as more strains are sequenced, we will be able to determine whether MAST-inferred strains are truly identical and if not, how much they differ in gene content and average nucleotide identity (ANI).

### Conclusions.

Overall, our results revealed that strain composition within the honey bee gut is complex despite the overall simplicity of the microbiome. Using the MAST approach, we were able to characterize the population dynamics of two host-associated microbes at a level not feasible with other methods. Future studies using controlled conditions (e.g., genotype, age, life history), will help reveal the factors that shape strain-level dynamics in the honey bee gut microbiome and provide answers to fundamental questions about the population genetics of host-associated microbiomes.

## MATERIALS AND METHODS

### Bee sampling and processing.

Bees were sampled from a single colony from each of the following locations: Texas, Tennessee, Florida, and Utah (see Table S1 in the supplemental material) and stored in 100% ethanol at 4°C. Bees from Texas were used in our previous study (6) and were sampled directly from the University of Texas (UT) Austin campus. Bees from Utah, Tennessee, and Florida were obtained from collaborators. Therefore, the sample size of bees from Texas was much larger than that of the other locations. The guts of all bees were dissected using sterile forceps and then homogenized. DNA was extracted using a cetyltrimethylammonium bromide (CTAB) bead-beating protocol described in reference 43. PCR was performed on the extracted DNA using the *guaA*, g*luS*, *pflA*, and *rimM* primers designed in reference 6 attached to Illumina adaptors. Triplicate 25-μl reactions were carried out with 0.25 μl Phusion high-fidelity DNA polymerase (New England Biolabs), 5 μl of Phusion HF buffer (New England Biolabs), 1.25 μl of dimethyl sulfoxide (DMSO), 1 μl (each) 10 μM primer, 14.5 μl H_2_O, and 1 μl of template DNA in buffer. The cycling conditions consisted of 98°C for 30 s, 25 cycles with 1 cycle consisting of 98°C for 10 s, 59°C for 30 s, and 72°C for 30 s, with a final extension at 72°C for 7 min. The amplicons were pooled and cleaned using AMPure XP beads (Beckman Coulter). The Genome Sequencing and Analysis Center at the University of Texas at Austin performed the barcoding and sequencing using Illumina MiSeq 2X300.

### Strain inference and identification of strain clusters.

Paired-end reads were merged together using FLASH v1.2.11 (44) with default parameters. Reads with low quality were discarded (average Phred score of <30), and the remaining reads were aligned against the reference sequence (*G. apicola* wkB1 or *S. alvi* wkB2) of each marker with BOWTIE2 v2.2.8 (45). Reads that aligned with gaps were excluded. Single nucleotide polymorphisms (SNPs) were identified, and each sequence was considered a potential strain. For each marker, a strain was inferred if present at >1% frequency in at least one sample (this threshold of 1% corresponds to an average of 460 reads per strain per sample). The total number of strains identified for each marker are provided in Table S2A. The number of strains identified in each sample are shown in Table S2B and the detailed description of all the strains is provided in Data Set S1 in the supplemental material.

10.1128/mSphere.00694-20.9 TABLE S2 (A) Number of strains detected for each marker. (B) Number of strains per sample (bee) for each marker. NA represents samples that reads were not obtained due to issues with DNA extraction or sequencing. Download Table S2, XLSX file, 0.01 MB. Copyright © 2020 Bobay et al. 2020 Bobay et al. This content is distributed under the terms of the Creative Commons Attribution 4.0 International license.

10.1128/mSphere.00694-20.10 DATA SET S1 Individual strain frequencies for all samples and makers. Download Data Set S1, XLSX file, 0.4 MB. Copyright © 2020 Bobay et al. 2020 Bobay et al. This content is distributed under the terms of the Creative Commons Attribution 4.0 International license.

Strain clusters were defined based on the frequency of strain co-occurrence within the same bee. For each pair of strains defined across the four markers, we defined a score *S* of co-occurrence as the number of bees containing both strains divided by the average number of bees containing either strain: $S=\frac{2b_{\mathrm{AB}}}{\left(b_{A}+b_{B}\right)}$, with *b_AB_* the number of bees containing both strains A and B, *b_A_* the number of bees containing strain A, and *b_B_* the number of bees containing strain B. These scores were used to build the heatmaps and the clusters with MCL v14-137 (46) with different inflation parameters: *I* = 1.2 (minimum), 2.0, 4.0, and 6.0. With *I* = 1.2, most strains were assembled into very few clusters (<20) composed of >30 strains per cluster. The three other inflation parameters yielded rather consistent clustering patterns—the number of clusters varied from 40 to 106 clusters across markers *guaA*, *gluS*, *pflA*, and *rimM* and inflation parameters (Fig. S7D). The different clustering parameters yielded similar results. We conducted the analysis with inflation parameter *I* = 2.0, since it yielded consistent numbers of clusters across gene markers (42 to 64 clusters for *guaA*, *gluS*, and *pflA*).

### Sequence analysis.

Unweighted UniFrac analysis was conducted with QIIME 1.9.1 (47) using the four gene markers *gluS*, *guaA*, *pflA*, and *rimM*. One sequence representative of each strain was selected to construct the phylogenetic tree of each marker. Each phylogenetic tree was then constructed using BIONJ (48) with the Jukes-Cantor model implemented in Seaview v4 (49). Analysis of reference genomes was conducted by downloading all reference genomes of *Gilliamella* available in GenBank (June 2019). The core genome was identified as follows. Briefly, orthologous genes were identified by pairwise genome comparison with Usearch Global (50). Two genes were considered orthologs when they showed ≥70% protein identity and ≥80% length conservation. Genes were then grouped into gene families by transitivity (i.e., two orthologous genes necessarily belong to the same family). Gene families with paralogs were excluded from the core genome. Additionally, gene families containing “double outliers” were excluded from the core genome. Double outliers were defined as sequences that present an abnormally low or high sequence identity score relative (i) to the other sequences of the gene family and (ii) to the average identity score of the other core genes of the corresponding genome. In both cases, outliers were defined as gene families containing at least one sequence with an identity score lower or higher than 1.5 × IQR (interquartile range) compared to (i) the other sequences of the gene family and (ii) to the average sequence identity of the other core genes of the corresponding genome. The scripts used to build the core genomes have been assembled into a computer package freely available on GitHub: https://github.com/lbobay/CoreCruncher. The sequences of each gene family were then aligned with MAFFT v7 (51) and back translated *in silico* into the nucleotide sequence of each corresponding genome. The different gene alignments were then merged into a single core genome concatenate.

### Phylogenies.

We used the entire set of genomes of Snodgrassella alvi and *Gilliamella* spp. from Apis mellifera to extract the four gene sequences *guaA*, g*luS*, *pflA*, and *rimM* from each genome, The sequences of each gene were aligned with MAFFT v7 (51), and the region of each marker was extracted. A representative sequence of each strain identified above was added to each marker alignment. Alignments were manually inspected, and no misaligned sequences were observed. For each marker alignment, phylogenetic trees were built with RAxML v8 (52) with a GAMMA + GTR model and 100 fast bootstrap replicates. The core genome phylogeny was run on the concatenate of the core genome using the same program and the same parameters.

### Simulations.

Simulations were initiated with the reference sequence of each marker. We simulated 144 sets of sequences to mimic the number of bees in our data set. For each bee, we simulated a number of sequences that closely matches the average number of distinct strains observed per bee (6 strains per bee for *guaA*, *gluS*, and *rimM*; 16 strains per bee for *pflA*). Each strain was generated following a Poisson process by introducing a number of SNPs that matches the average number of SNPs observed across strains found in the same bee (Fig. 1). Simulations were conducted with *CoreSimul* (53) either by introducing SNPs randomly along each sequence to test the impact of PCR artifacts or by introducing SNPs with different probabilities across codon positions to test the effect of purifying sequence on strain inference. The relative probability of substitutions across codon positions was defined empirically based on the distribution of observed SNPs across the distinct strains inferred by each marker: 0.12, 0.10, and 0.78 for *guaA*, 0.17, 0.04, and 0.79 for *gluS*, 0.14, 0.04, and 0.82 for *pflA*, and 0.20, 0.09, and 0.71 for *rimM* at the first, second, and third codon positions, respectively. The scripts used for the simulations are available at https://github.com/lbobay/CoreSimul.

### Data availability.

All raw sequencing reads are deposited in NCBI Sequence Read Bioproject under accession numbers PRJNA562505 and PRJNA415093. All other data generated are included in the supplemental material files.
