# Supplementary material for: Strain Structure and Dynamics Revealed by Targeted Deep Sequencing of the Honey Bee Gut Microbiome
Source: mSphere. 2020 Aug 26;5(4):e00694-20. doi: 10.1128/mSphere.00694-20 (PMC7449624; doi:10.1128/mSphere.00694-20)
Supplement: FIG S1 [file mSphere.00694-20-sf001.pdf]

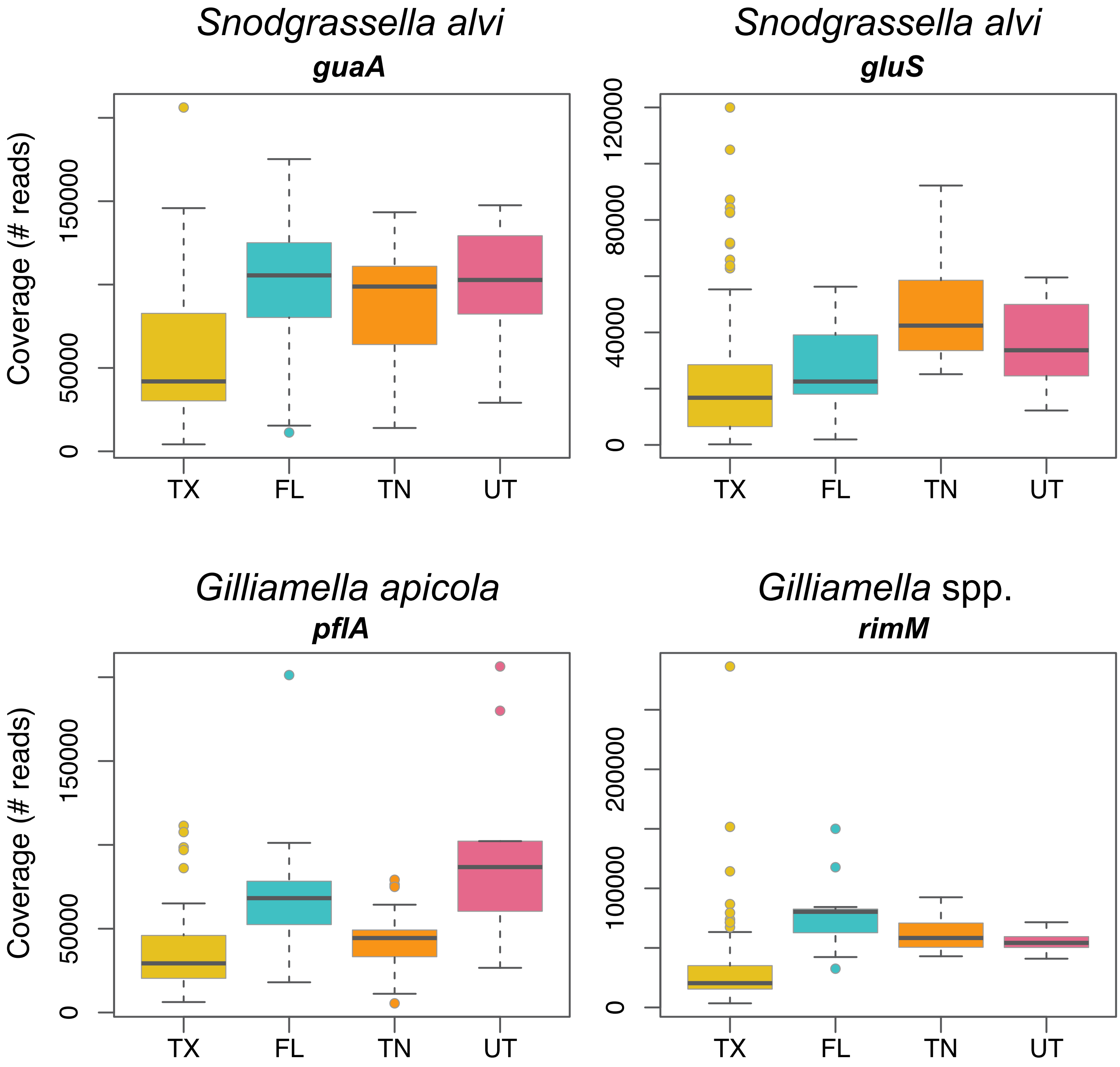

Snodgrassella alvi
guaA
Coverage (# reads)
0
50000
150000
TX
FL
TN
UT
Snodgrassella alvi
gluS
0
40000
80000
120000
TX
FL
TN
UT
Gilliamella apicola
pflA
Coverage (# reads)
0
50000
150000
TX
FL
TN
UT
Gilliamella spp.
rimM
0
100000
200000
TX
FL
TN
UT
