# Supplementary material for: Strain Structure and Dynamics Revealed by Targeted Deep Sequencing of the Honey Bee Gut Microbiome
Source: mSphere. 2020 Aug 26;5(4):e00694-20. doi: 10.1128/mSphere.00694-20 (PMC7449624; doi:10.1128/mSphere.00694-20)
Supplement: FIG S2 [file mSphere.00694-20-sf002.pdf]

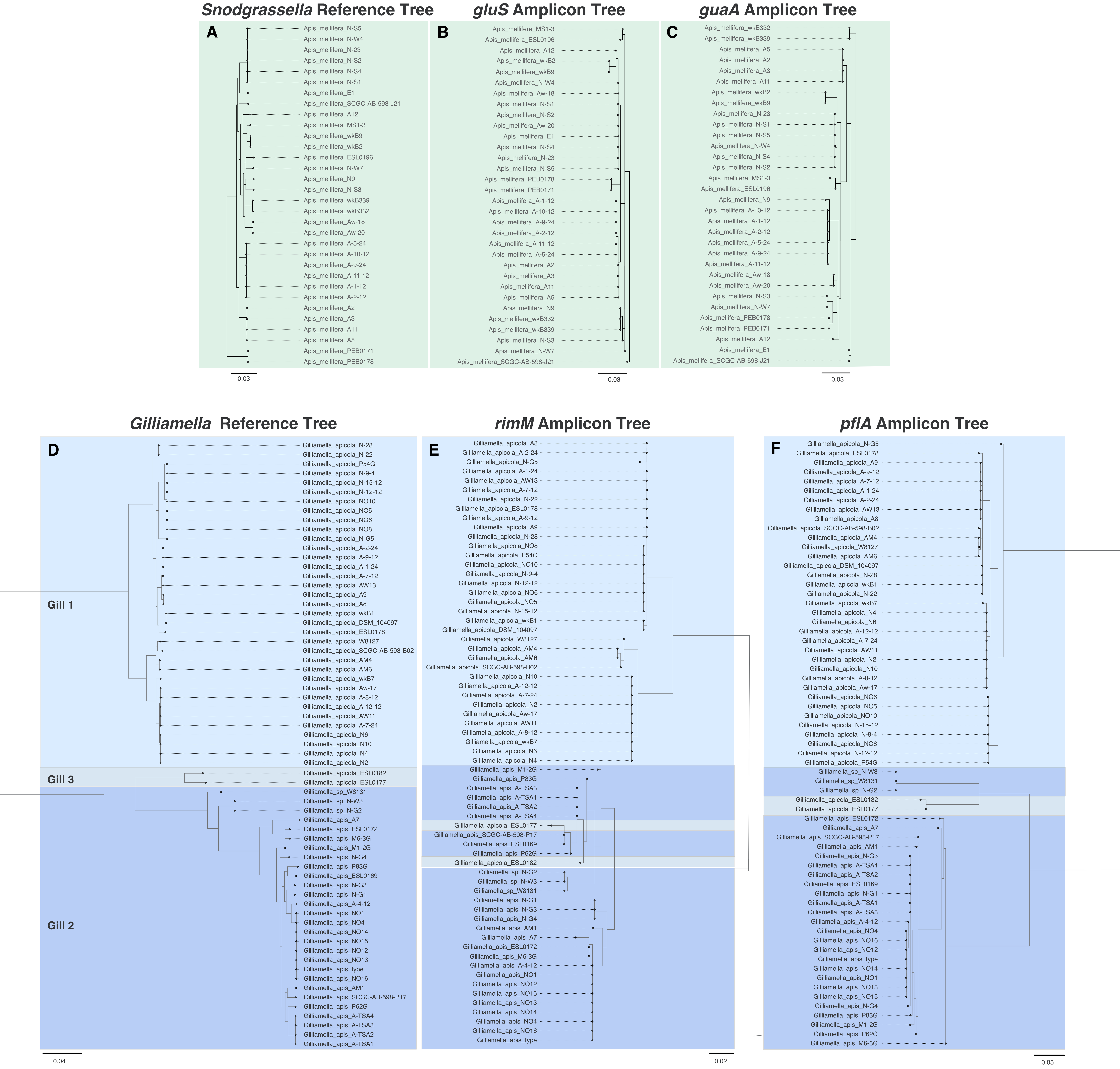

Snodgrassella Reference Tree
gluS Amplicon Tree
guaA Amplicon Tree
A
Apis_mellifera_N-S5
Apis_mellifera_N-W4
Apis_mellifera_N-23
Apis_mellifera_N-S2
Apis_mellifera_N-S4
Apis_mellifera_N-S1
Apis_mellifera_E1
Apis_mellifera_SCGC-AB-598-J21
Apis_mellifera_A12
Apis_mellifera_MS1-3
Apis_mellifera_wkB9
Apis_mellifera_wkB2
Apis_mellifera_ESL0196
Apis_mellifera_N-W7
Apis_mellifera_N9
Apis_mellifera_N-S3
Apis_mellifera_wkB339
Apis_mellifera_wkB332
Apis_mellifera_Aw-18
Apis_mellifera_Aw-20
Apis_mellifera_A-5-24
Apis_mellifera_A-10-12
Apis_mellifera_A-9-24
Apis_mellifera_A-11-12
Apis_mellifera_A-1-12
Apis_mellifera_A-2-12
Apis_mellifera_A2
Apis_mellifera_A3
Apis_mellifera_A11
Apis_mellifera_A5
Apis_mellifera_PEB0171
Apis_mellifera_PEB0178
0.03
B
Apis_mellifera_MS1-3
Apis_mellifera_ESL0196
Apis_mellifera_A12
Apis_mellifera_wkB2
Apis_mellifera_wkB9
Apis_mellifera_N-W4
Apis_mellifera_Aw-18
Apis_mellifera_N-S1
Apis_mellifera_N-S2
Apis_mellifera_Aw-20
Apis_mellifera_E1
Apis_mellifera_N-S4
Apis_mellifera_N-23
Apis_mellifera_N-S5
Apis_mellifera_PEB0178
Apis_mellifera_PEB0171
Apis_mellifera_A-1-12
Apis_mellifera_A-10-12
Apis_mellifera_A-9-24
Apis_mellifera_A-2-12
Apis_mellifera_A-11-12
Apis_mellifera_A-5-24
Apis_mellifera_A2
Apis_mellifera_A3
Apis_mellifera_A11
Apis_mellifera_A5
Apis_mellifera_N9
Apis_mellifera_wkB332
Apis_mellifera_wkB339
Apis_mellifera_N-S3
Apis_mellifera_N-W7
Apis_mellifera_SCGC-AB-598-J21
0.03
C
Apis_mellifera_wkB332
Apis_mellifera_wkB339
Apis_mellifera_A5
Apis_mellifera_A2
Apis_mellifera_A3
Apis_mellifera_A11
Apis_mellifera_wkB2
Apis_mellifera_wkB9
Apis_mellifera_N-23
Apis_mellifera_N-S1
Apis_mellifera_N-S5
Apis_mellifera_N-W4
Apis_mellifera_N-S4
Apis_mellifera_N-S2
Apis_mellifera_MS1-3
Apis_mellifera_ESL0196
Apis_mellifera_N9
Apis_mellifera_A-10-12
Apis_mellifera_A-1-12
Apis_mellifera_A-2-12
Apis_mellifera_A-5-24
Apis_mellifera_A-9-24
Apis_mellifera_A-11-12
Apis_mellifera_Aw-18
Apis_mellifera_Aw-20
Apis_mellifera_N-S3
Apis_mellifera_N-W7
Apis_mellifera_PEB0178
Apis_mellifera_PEB0171
Apis_mellifera_A12
Apis_mellifera_E1
Apis_mellifera_SCGC-AB-598-J21
0.03
Gilliamella Reference Tree
rimM Amplicon Tree
pflA Amplicon Tree
D
Gill 1
Gill 3
Gill 2
Gilliamella_apicola_N-28
Gilliamella_apicola_N-22
Gilliamella_apicola_P54G
Gilliamella_apicola_N-9-4
Gilliamella_apicola_N-15-12
Gilliamella_apicola_N-12-12
Gilliamella_apicola_NO10
Gilliamella_apicola_NO5
Gilliamella_apicola_NO6
Gilliamella_apicola_NO8
Gilliamella_apicola_N-G5
Gilliamella_apicola_A-2-24
Gilliamella_apicola_A-9-12
Gilliamella_apicola_A-1-24
Gilliamella_apicola_A-7-12
Gilliamella_apicola_AW13
Gilliamella_apicola_A9
Gilliamella_apicola_A8
Gilliamella_apicola_wkB1
Gilliamella_apicola_DSM_104097
Gilliamella_apicola_ESL0178
Gilliamella_apicola_W8127
Gilliamella_apicola_SCGC-AB-598-B02
Gilliamella_apicola_AM4
Gilliamella_apicola_AM6
Gilliamella_apicola_wkB7
Gilliamella_apicola_Aw-17
Gilliamella_apicola_A-8-12
Gilliamella_apicola_A-12-12
Gilliamella_apicola_AW11
Gilliamella_apicola_A-7-24
Gilliamella_apicola_N6
Gilliamella_apicola_N10
Gilliamella_apicola_N4
Gilliamella_apicola_N2
Gilliamella_apicola_ESL0182
Gilliamella_apicola_ESL0177
Gilliamella_sp_W8131
Gilliamella_sp_N-W3
Gilliamella_sp_N-G2
Gilliamella_apis_A7
Gilliamella_apis_ESL0172
Gilliamella_apis_M6-3G
Gilliamella_apis_M1-2G
Gilliamella_apis_N-G4
Gilliamella_apis_P83G
Gilliamella_apis_ESL0169
Gilliamella_apis_N-G3
Gilliamella_apis_N-G1
Gilliamella_apis_A-4-12
Gilliamella_apis_NO1
Gilliamella_apis_NO4
Gilliamella_apis_NO14
Gilliamella_apis_NO15
Gilliamella_apis_NO12
Gilliamella_apis_NO13
Gilliamella_apis_type
Gilliamella_apis_NO16
Gilliamella_apis_AM1
Gilliamella_apis_SCGC-AB-598-P17
Gilliamella_apis_P62G
Gilliamella_apis_A-TSA4
Gilliamella_apis_A-TSA3
Gilliamella_apis_A-TSA2
Gilliamella_apis_A-TSA1
0.04
E
Gilliamella_apicola_A8
Gilliamella_apicola_A-2-24
Gilliamella_apicola_N-G5
Gilliamella_apicola_A-1-24
Gilliamella_apicola_AW13
Gilliamella_apicola_A-7-12
Gilliamella_apicola_N-22
Gilliamella_apicola_ESL0178
Gilliamella_apicola_A-9-12
Gilliamella_apicola_A9
Gilliamella_apicola_N-28
Gilliamella_apicola_NO8
Gilliamella_apicola_P54G
Gilliamella_apicola_NO10
Gilliamella_apicola_N-9-4
Gilliamella_apicola_N-12-12
Gilliamella_apicola_NO6
Gilliamella_apicola_NO5
Gilliamella_apicola_N-15-12
Gilliamella_apicola_wkB1
Gilliamella_apicola_DSM_104097
Gilliamella_apicola_W8127
Gilliamella_apicola_AM4
Gilliamella_apicola_AM6
Gilliamella_apicola_SCGC-AB-598-B02
Gilliamella_apicola_N10
Gilliamella_apicola_A-12-12
Gilliamella_apicola_A-7-24
Gilliamella_apicola_N2
Gilliamella_apicola_Aw-17
Gilliamella_apicola_AW11
Gilliamella_apicola_A-8-12
Gilliamella_apicola_wkB7
Gilliamella_apicola_N6
Gilliamella_apicola_N4
Gilliamella_apis_M1-2G
Gilliamella_apis_P83G
Gilliamella_apis_A-TSA3
Gilliamella_apis_A-TSA1
Gilliamella_apis_A-TSA2
Gilliamella_apis_A-TSA4
Gilliamella_apicola_ESL0177
Gilliamella_apis_SCGC-AB-598-P17
Gilliamella_apis_ESL0169
Gilliamella_apis_P62G
Gilliamella_apicola_ESL0182
Gilliamella_sp_N-G2
Gilliamella_sp_N-W3
Gilliamella_sp_W8131
Gilliamella_apis_N-G1
Gilliamella_apis_N-G3
Gilliamella_apis_N-G4
Gilliamella_apis_AM1
Gilliamella_apis_A7
Gilliamella_apis_ESL0172
Gilliamella_apis_M6-3G
Gilliamella_apis_A-4-12
Gilliamella_apis_NO1
Gilliamella_apis_NO12
Gilliamella_apis_NO15
Gilliamella_apis_NO13
Gilliamella_apis_NO14
Gilliamella_apis_NO4
Gilliamella_apis_NO16
Gilliamella_apis_type
0.02
F
Gilliamella_apicola_N-G5
Gilliamella_apicola_ESL0178
Gilliamella_apicola_A9
Gilliamella_apicola_A-9-12
Gilliamella_apicola_A-7-12
Gilliamella_apicola_A-1-24
Gilliamella_apicola_A-2-24
Gilliamella_apicola_AW13
Gilliamella_apicola_A8
Gilliamella_apicola_SCGC-AB-598-B02
Gilliamella_apicola_AM4
Gilliamella_apicola_W8127
Gilliamella_apicola_AM6
Gilliamella_apicola_DSM_104097
Gilliamella_apicola_N-28
Gilliamella_apicola_wkB1
Gilliamella_apicola_N-22
Gilliamella_apicola_wkB7
Gilliamella_apicola_N4
Gilliamella_apicola_N6
Gilliamella_apicola_A-12-12
Gilliamella_apicola_A-7-24
Gilliamella_apicola_AW11
Gilliamella_apicola_N2
Gilliamella_apicola_N10
Gilliamella_apicola_A-8-12
Gilliamella_apicola_Aw-17
Gilliamella_apicola_NO6
Gilliamella_apicola_NO5
Gilliamella_apicola_NO10
Gilliamella_apicola_N-15-12
Gilliamella_apicola_N-9-4
Gilliamella_apicola_NO8
Gilliamella_apicola_N-12-12
Gilliamella_apicola_P54G
Gilliamella_sp_N-W3
Gilliamella_sp_W8131
Gilliamella_sp_N-G2
Gilliamella_apicola_ESL0182
Gilliamella_apicola_ESL0177
Gilliamella_apis_ESL0172
Gilliamella_apis_A7
Gilliamella_apis_SCGC-AB-598-P17
Gilliamella_apis_AM1
Gilliamella_apis_N-G3
Gilliamella_apis_A-TSA4
Gilliamella_apis_A-TSA2
Gilliamella_apis_ESL0169
Gilliamella_apis_N-G1
Gilliamella_apis_A-TSA1
Gilliamella_apis_A-TSA3
Gilliamella_apis_A-4-12
Gilliamella_apis_NO4
Gilliamella_apis_NO16
Gilliamella_apis_NO12
Gilliamella_apis_type
Gilliamella_apis_NO14
Gilliamella_apis_NO1
Gilliamella_apis_NO13
Gilliamella_apis_NO15
Gilliamella_apis_N-G4
Gilliamella_apis_P83G
Gilliamella_apis_M1-2G
Gilliamella_apis_P62G
Gilliamella_apis_M6-3G
0.05
