# Supplementary figures and images for: Strain Structure and Dynamics Revealed by Targeted Deep Sequencing of the Honey Bee Gut Microbiome
Source: mSphere. 2020 Aug 26;5(4):e00694-20. doi: 10.1128/mSphere.00694-20 (PMC7449624; doi:10.1128/mSphere.00694-20)

***gluS***

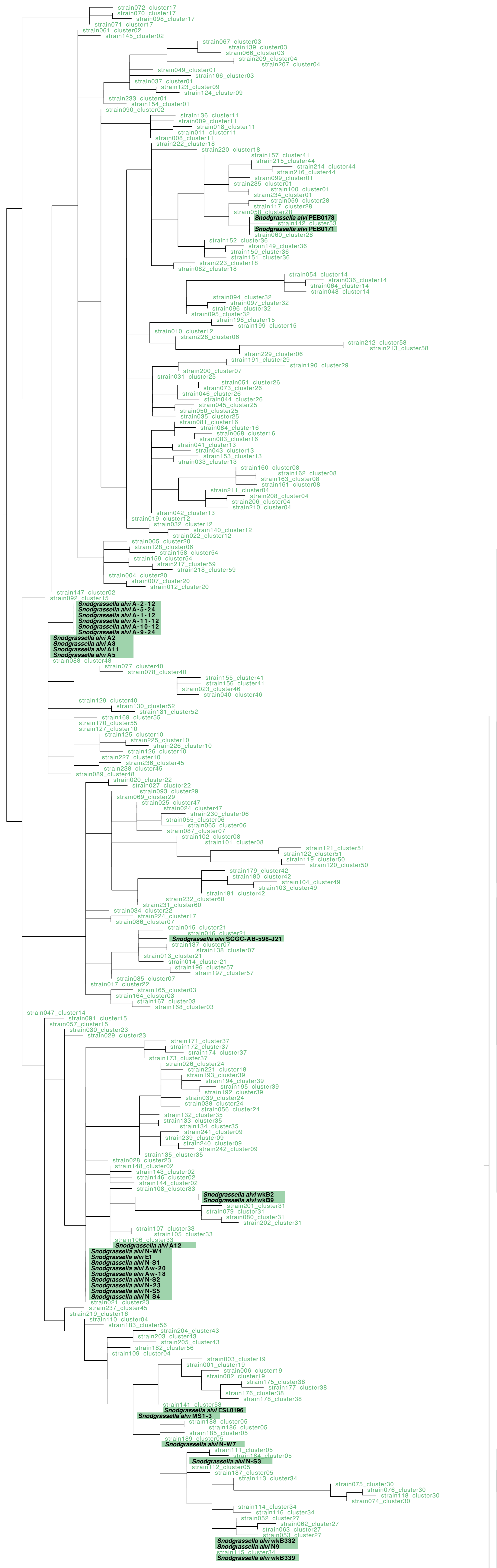

0.2

***guaA***

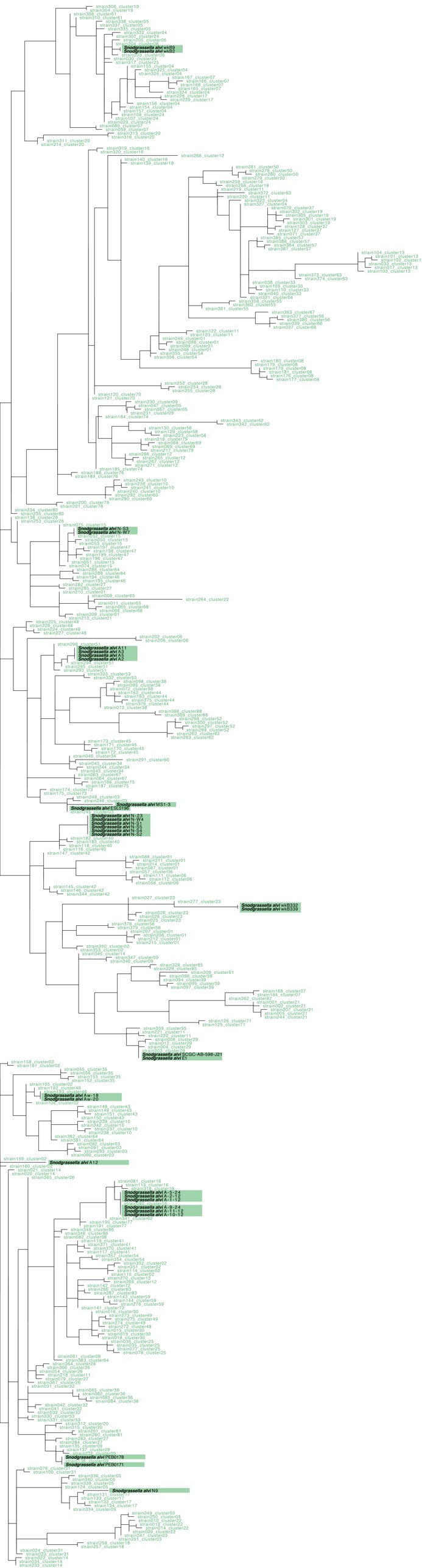

0.7

Supplement: FIG S3 [file mSphere.00694-20-sf003.pdf]

rimM

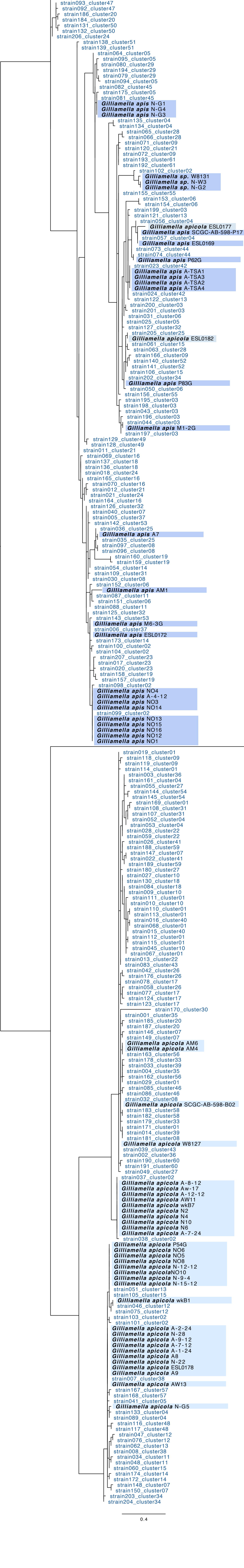

0.4

pflA

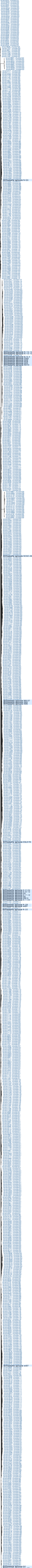

0.4

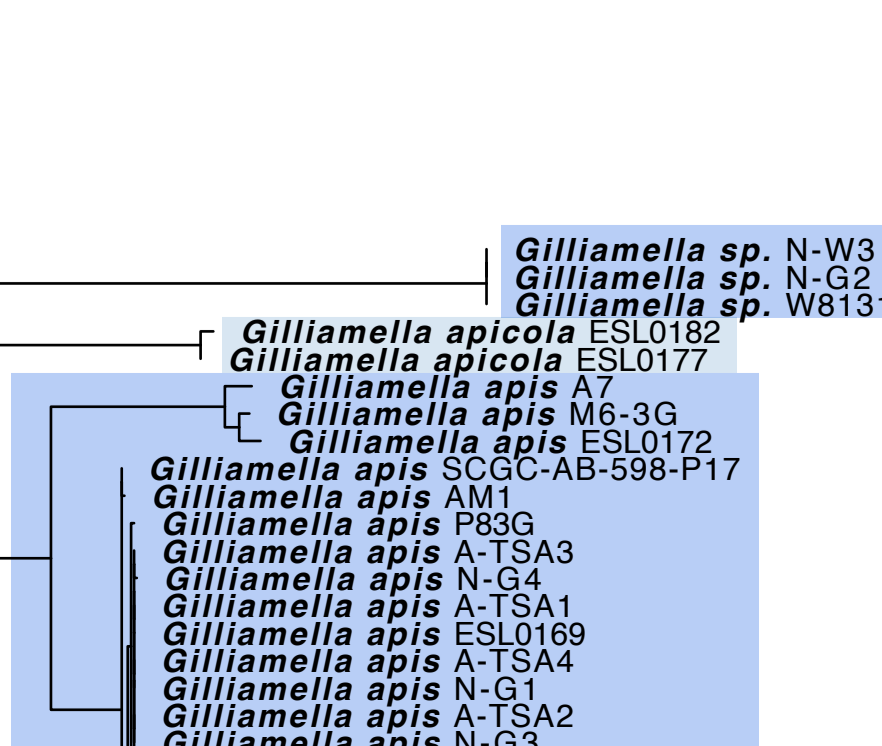

0.4

Supplement: FIG S4 [file mSphere.00694-20-sf004.pdf]

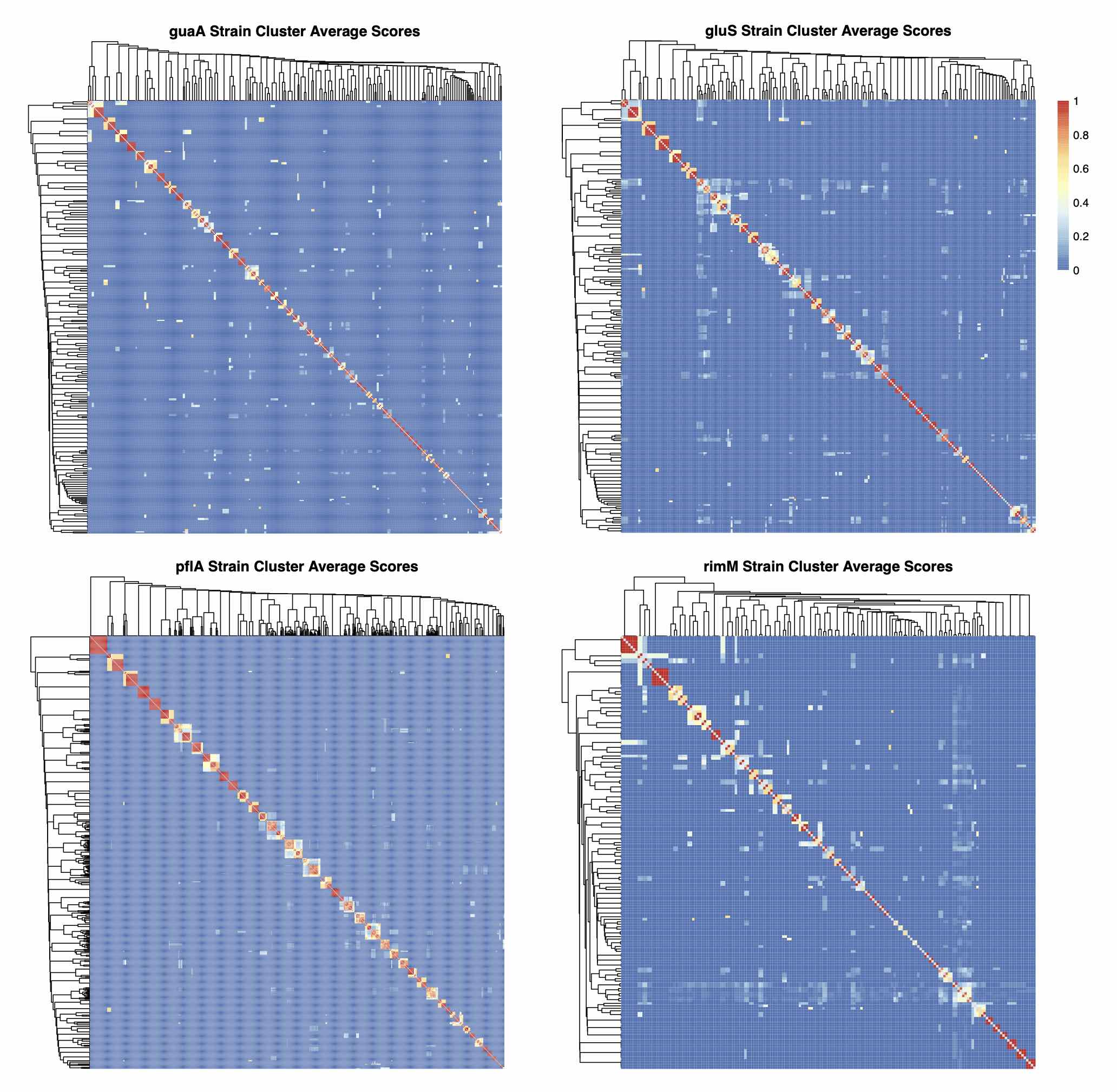

Supplement: FIG S6 [file mSphere.00694-20-sf006.jpg]

**A**

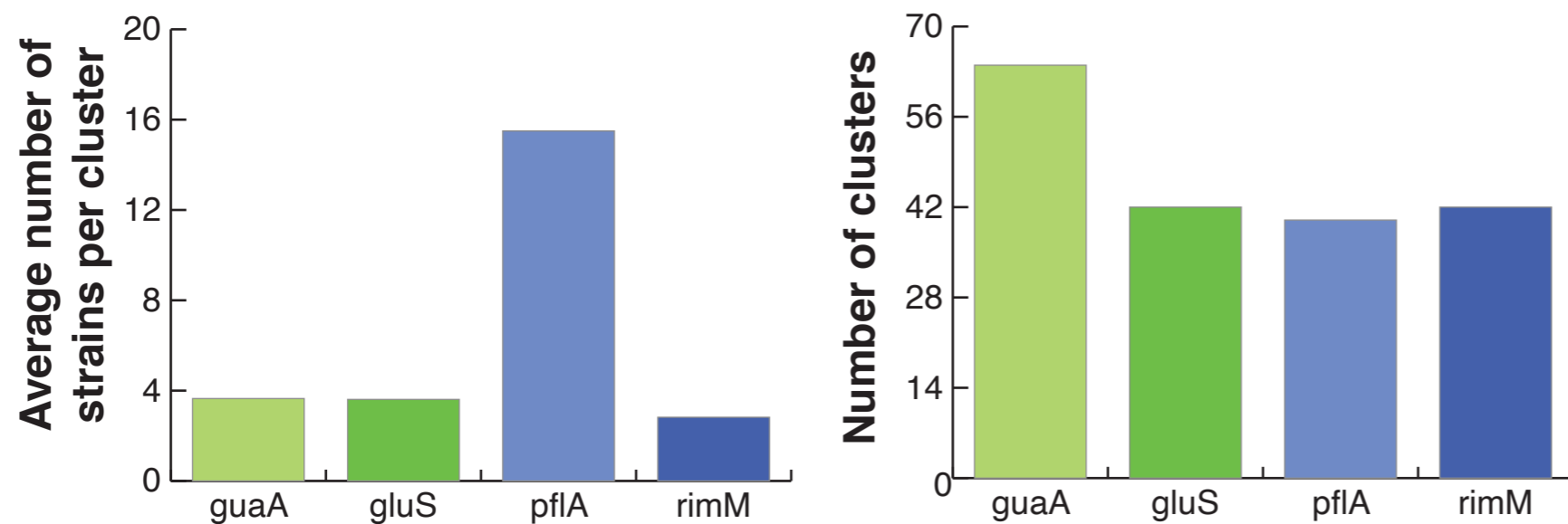

**B**

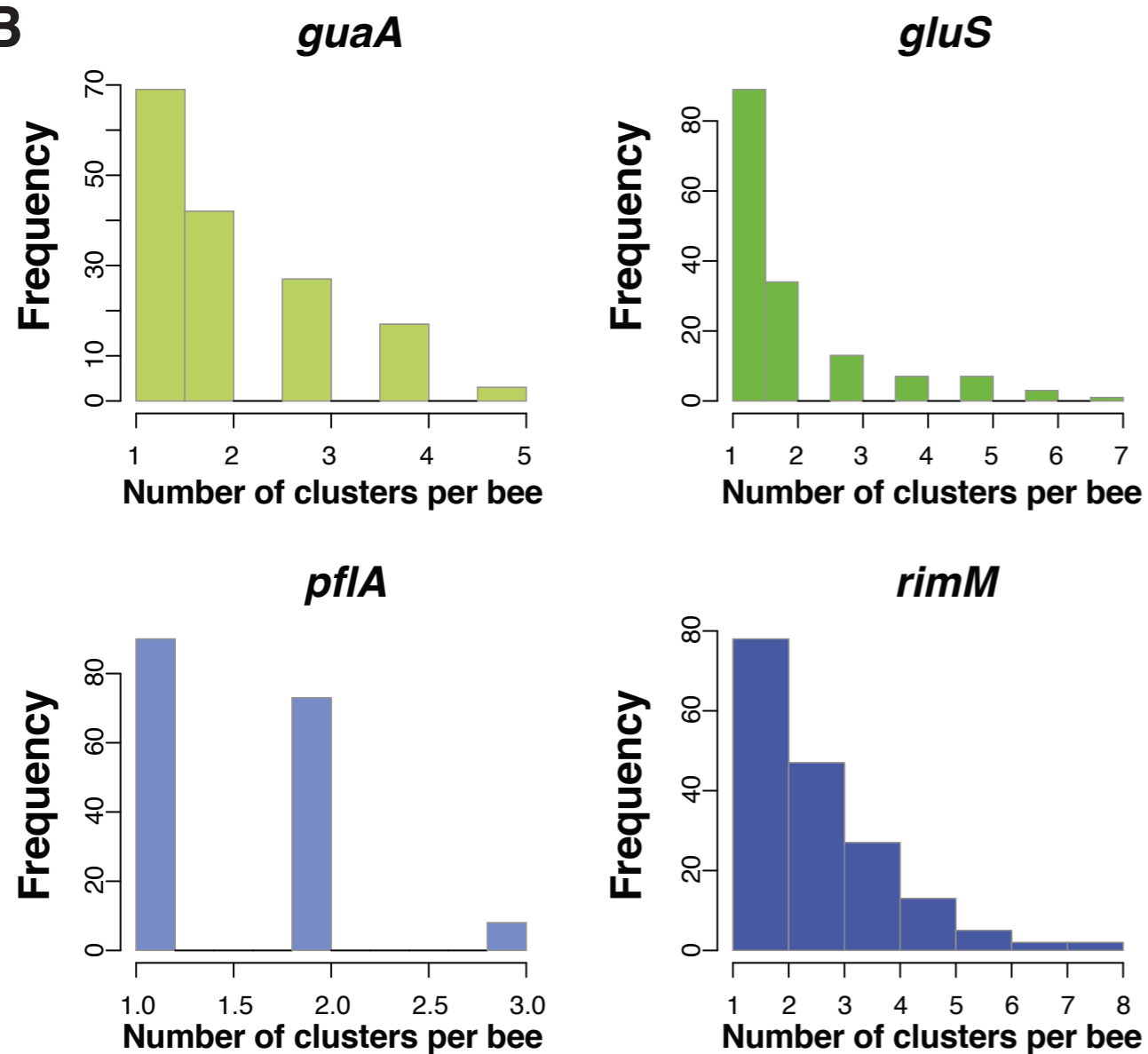

**C**

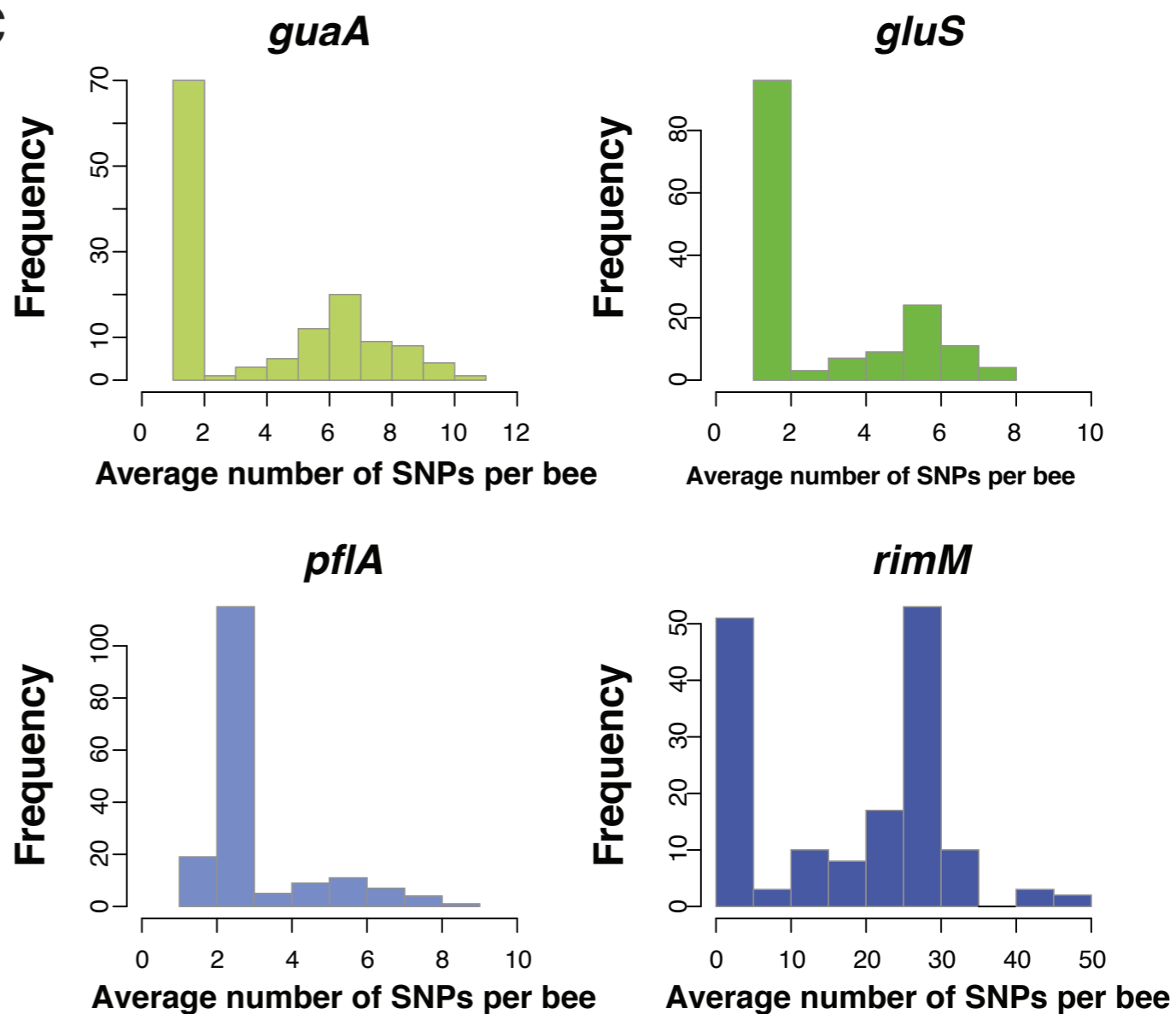

**D**

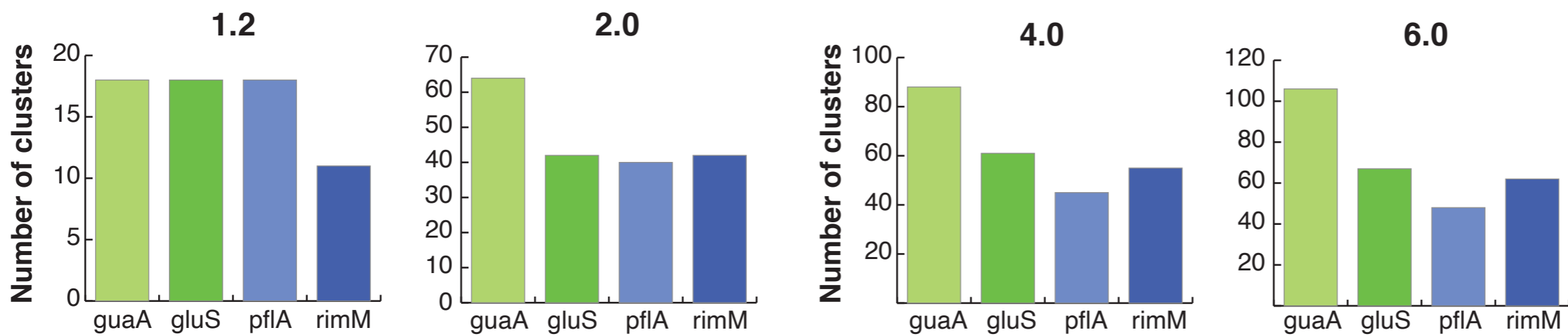

Supplement: FIG S7 [file mSphere.00694-20-sf007.pdf]
