# Supplementary material for: Strain Structure and Dynamics Revealed by Targeted Deep Sequencing of the Honey Bee Gut Microbiome
Source: mSphere. 2020 Aug 26;5(4):e00694-20. doi: 10.1128/mSphere.00694-20 (PMC7449624; doi:10.1128/mSphere.00694-20)
Supplement: FIG S5 [file mSphere.00694-20-sf005.pdf]

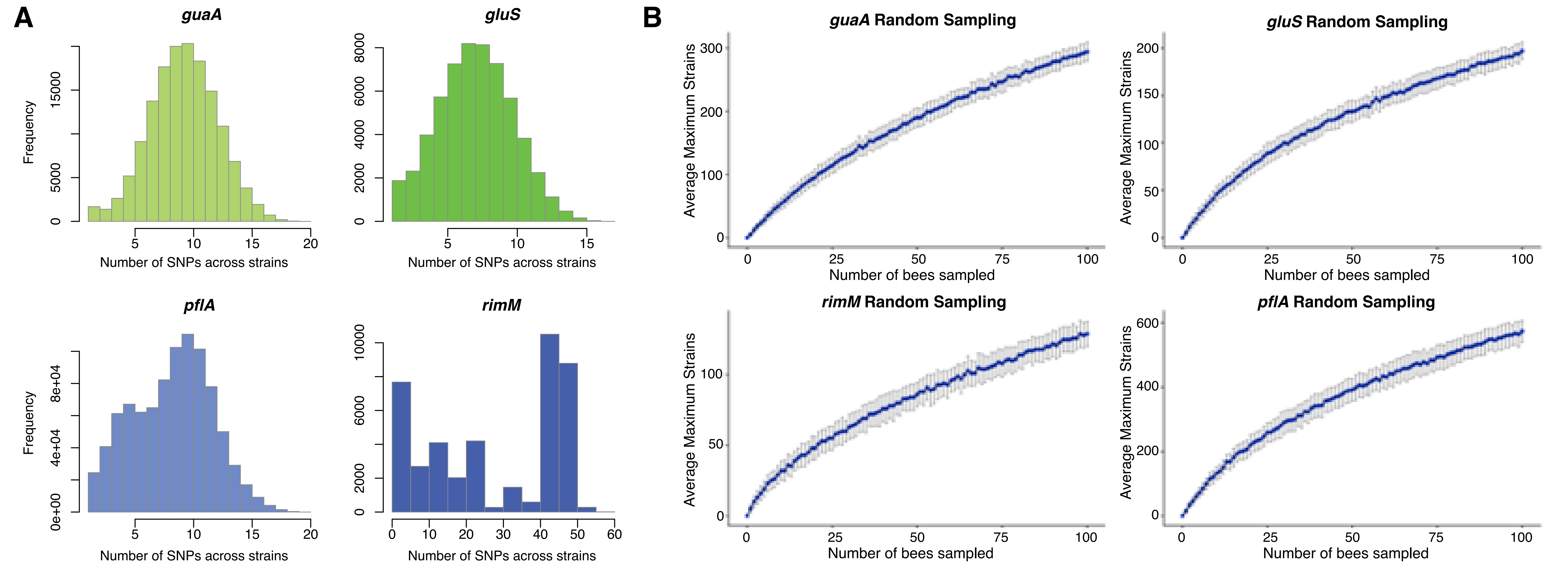

A
guaA
gluS
pflA
rimM
Frequency
Number of SNPs across strains
B
guaA Random Sampling
gluS Random Sampling
rimM Random Sampling
pflA Random Sampling
Average Maximum Strains
Number of bees sampled
